# Do morphokinetic data sets inform pregnancy potential?

**DOI:** 10.1007/s10815-016-0649-9

**Published:** 2016-02-03

**Authors:** Robert Milewski, Anna Justyna Milewska, Agnieszka Kuczyńska, Bożena Stankiewicz, Waldemar Kuczyński

**Affiliations:** Department of Statistics and Medical Informatics, Medical University of Bialystok, Szpitalna 37, 15-295 Bialystok, Poland; Centre for Reproductive Medicine KRIOBANK, Bialystok, Poland; Department of Reproduction and Gynecological Endocrinology, Medical University of Bialystok, Bialystok, Poland; Department of Gynecology, Medical University of Bialystok, Bialystok, Poland

**Keywords:** Infertility, IVF ET, Morphokinetic parameters, Embryoscope, Time-lapse recordings, Principal component analysis

## Abstract

**Purpose:**

The aim of this study was to create a model to predict the implantation of transferred embryos based on information contained in the morphokinetic parameters of time-lapse monitoring.

**Methods:**

An analysis of time-lapse recordings of 410 embryos transferred in 343 cycles of in vitro fertilization (IVF) treatment was performed. The study was conducted between June 2012 and November 2014. For each embryo, the following data were collected: the duration of time from the intracytoplasmic sperm injection (ICSI) procedure to further division for two, three, four, and five blastomeres, time intervals between successive divisions, and the level of fragmentation assessed in successive time-points. Principal component analysis (PCA) and logistic regression were used to create a predictive model.

**Results:**

Based on the results of principal component analysis and logistic regression analysis, a predictive equation was constructed. Statistically significant differences (*p* < 0.001) in the size of the created parameter between the implanted group (the median value: Me = −5.18 and quartiles: *Q*_1_ = −5.61; *Q*_3_ = −4.79) and the non-implanted group (Me = −5.69, *Q*_1_ = −6.34; *Q*_3_ = −5.16) were found. A receiver operating characteristic (ROC) curve constructed for the considered model showed the good quality of this predictive equation. The area under the ROC curve was AUC = 0.70 with a 95 % confidence interval (0.64, 0.75). The presented model has been validated on an independent data set, illustrating that the model is reliable and repeatable.

**Conclusions:**

Morphokinetic parameters contain information useful in the process of creating pregnancy prediction models. However, embryo quality is not the only factor responsible for implantation, and, thus, the power of prediction of the considered model is not as high as in models for blastocyst formation. Nevertheless, as illustrated by the results of this study, the application of advanced data-mining methods in reproductive medicine allows one to create more accurate and useful models.

## Introduction

Progress in reproductive medicine has resulted in a growth in the efficacy of infertility treatment. The pregnancy rate has increased in recent years to over 40 % [1]. The ability to assess the developmental potential of embryos cultured has a crucial impact on the effectiveness of infertility treatment. However, the scoring of embryo development is restricted if based on static observation only. The application of time-lapse imaging is leading to new possibilities for the development of scoring systems. It may be time to create new selection strategies based on the information gained through the use of time-lapse imaging [2]. Aparicio et al. claim that important improvements in embryo selection may be realized using time-lapse technology because of the possibility of selecting viable embryos with a high developmental potential [3]. Cetinkaya et al. present an itemized comparative analysis of kinetic parameters focusing on relative time ratios and time intervals [4]. They produced an equation based on analyzed morphokinetic variables, allowing for the prediction of blastocyst formation. The use of a time-lapse monitoring system is related to better reproductive outcomes in comparison with conventional methods [5]. This technology offers the opportunity to continuously observe embryo development, delivering a non-invasive method to enhance the precision of information acquired [6]. Morphokinetic predictive markers are created in such a way that endows them with the potential to improve the effectiveness of embryo selection, finally allowing single embryo transfers to be performed, thereby minimizing the rate of multiple pregnancies without the loss of treatment efficacy. However, Racowsky et al. [7] pay attention to the limitations of studies reporting algorithms that may assist in selecting the most viable embryos. They list variables other than embryo health (e.g., the type of ovarian stimulation or culture conditions) that influence the timing of embryo development. Therefore, in their assessment, created scoring systems should not be limited to time-lapse parameters only. They also claim that a lack of universally accepted nomenclature for morphokinetic features limits the ability to compare results among different studies. In addition to developing a universally accepted nomenclature, studies with the aim of constructing and validating scoring systems that depend on morphologic as well as kinetic features, in order to utilize time-lapse systems to the best advantage, are greatly needed.

Despite Racowsky et al.’s concerns, many predictive models for blastocyst formation based on morphokinetic parameters and time-lapse evaluation have been proposed. Cruz et al. [8] have shown a hierarchical model differentiating embryos based on development to blastocyst stage rate. They emphasize that time-lapse evaluation of early events in embryo development is a hopeful tool for the prediction of achievement of the blastocyst stage. Milewski et al. [9] have used a different approach, presenting an algorithm built on the basis of selected absolute and relative morphokinetic parameters. As a result, the authors concluded that there is a need for the construction of a similar model to predict the implantation of transferred embryos. To date, not many such models have been proposed. Furthermore, those which have been presented do not have sufficiently high predictive powers.

That being the case, the aim of this study was to create a model to predict the implantation of transferred embryos based on information contained in morphokinetic parameters of time-lapse monitoring.

## Materials and methods

An analysis of time-lapse recordings of 410 embryos transferred in 343 cycles of in vitro fertilization (IVF) treatment was performed. The study was carried out in the Centre for Reproductive Medicine Kriobank in Bialystok, Poland, between June 2012 and November 2014. All embryos were obtained after fertilization according to the standard intracytoplasmic sperm injection (ICSI) procedure. Most embryos were cultured to the blastocyst stage; only about 20 % of them were transferred on the second or third day of culture. In most cases (276 transfers), a single embryo transfer (SET) was conducted. Using this kind of transfer, there were 109 implantations, which accounted for about 39.5 % of all SET procedures. In other treatments, two embryos were transferred (134 embryos in 67 cycles), among which 65 cycles resulted in a lack of implantation and, in two cycles, the implantation of both embryos. In treatments with multiple transfers, but only single implantation, it was not possible to determine which of the transferred embryos had implanted. Such cases were not included in the analysis. No other exclusion criteria were applied; the data of all patients treated within a specific time period were included in the analysis. Among all analyzed embryos, the implantation rate (per embryo) amounted to approximately 27.6 %. The implantation of transferred embryos was confirmed at an ultrasound scanning for gestational sacs.

The embryo-slide for the time-lapse system consisted of 12 cylindrical wells, each holding a culture medium droplet of about 20 μl of Quinn’s Advantage Protein Plus Cleavage Medium (SAGE, USA). The wells were covered with mineral oil (SAGE, USA) to prevent evaporation. Embryos were placed individually into the wells, and then slides were placed in the Embryoscope. The culture parameters were set at 37.0 °C, 5.0 % CO_2_, and 5.0 % O_2_. The images of each embryo were acquired every 7 min at five different focal planes to enable accurate assessment of embryo morphology.

**Fig. 1:**
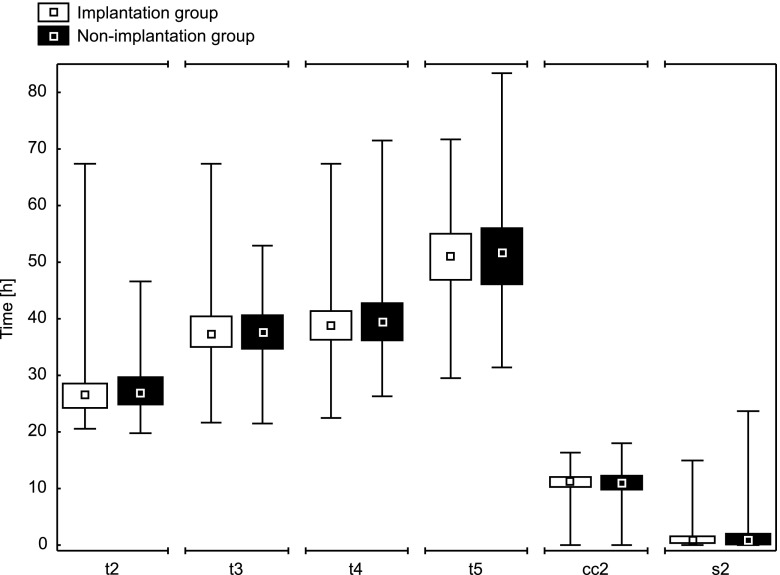
Distribution of morphokinetic parameters for implanted and non-implanted groups of embryos (median, quartiles, and min-max)

For each embryo, absolute morphokinetic parameters *t*2, *t*3, *t*4, and *t*5 (times from ICSI fertilization to further divisions into two, three, four, and five blastomeres), and relative morphokinetic parameters cc2 = *t*3−*t*2 and *s*2 = *t*4−*t*3 (intervals between successive divisions) were collected. Descriptive statistics (median, quartiles, and range) for absolute and relative morphokinetic parameters in implanted and non-implanted groups of embryos are presented below in a box-and-whisker plot (Fig. 1). Additionally, the levels of fragmentation assessed in *t*2, *t*3, *t*4, and *t*5 time-points were collected as listed respectively: fr2, fr3, fr4, and fr5. These parameters have an ordinal nature. They can take the values 1–4, which represent compartments of fragmentation percentage, respectively: (0–10 %), (10–20 %), (20–50 %), and (50–100 %). The women’s age range was 22–47 years, with the median value of 34 years (*Q*_1_ = 31 years; *Q*_3_ = 36 years).

The algorithm used in [9] to create the predictive model for blastocyst formation was applied here to construct a model that predicts implantation. The absolute and relative morphokinetic parameters were divided into four groups called C1–C4, with respect to the median value and quartiles (first and third). The pregnancy rates for each parameter in each group were determined. On the basis of such rates, an “individual score” (the value 0, 1, or 2) was assigned to each C1–C4 compartment. Then, a logistic regression analysis was conducted, where the individual scores were used as independent variables and implantation as a dependent variable. The parameters of the chosen multivariate logistic regression model were used to create the Sc score, which can be treated as a predictor of implantation.

The second predictive model for implantation was constructed on the basis of principal component analysis (PCA) and logistic regression analysis. The PCA technique is a data-mining method that relies on the transformation of an initial correlated set of features into new uncorrelated variables called principal components. This method is applicable to regression analysis when it is impossible to include all variables in a model because of multicollinearity. The solution in such a situation is to replace them with principal components [10].

Because the analyzed morphokinetic parameters (except *s*2) do not have a monotonic character (very low as well as very high values are not good predictors of implantation), the assumption presented in [9] that the values of the parameter most favorable to pregnancy will be close to the median value in the “successful” group of embryos was applied. Therefore, for all morphokinetic parameters (except *s*2), the distance between the parameter value and the median value in the group of implanted embryos (called, respectively, *t*2_*m*_, *t*3_*m*_, *t*4_*m*_, *t*5_*m*_, and cc2_*m*_) was calculated according to the formula:

$$ {t}_m=\left|t-\mathrm{Median}(t)\right| $$

The PCA method requires the standardization of variables according to the mean and standard deviation (SD) values [10]. Therefore, for all *t*_*m*_ variables, as well as for *s*2, standardized parameters were created and called *t*2_st_, *t*3_st_, *t*4_st_, *t*5_st_, cc2_st_, and *s*2_st_, according to the formula:

$$ {t}_{\mathrm{st}}=\left({t}_m-\mathrm{Mean}\left({t}_m\right)\right)/\mathrm{S}\mathrm{D}\left({t}_m\right) $$

For the *s*2 variable in the place of *s*2_*m*_, the original *s*2 parameter was substituted (this is the only monotonic morphokinetic parameter).

After the application of the PCA algorithm, the matrix of coefficients *α*_nm_ was generated. Based on this matrix, six principal components *f*_*n*_ were calculated, as a linear combination of standardized *t*_st_ parameters:

$$ {f}_n={\upalpha}_{n\;t{2}_{\mathrm{st}}}\ast t{2}_{\mathrm{st}}+{\upalpha}_{n\;t{3}_{\mathrm{st}}}\ast t{3}_{\mathrm{st}}+{\upalpha}_{n\;t{4}_{\mathrm{st}}}\ast t{4}_{\mathrm{st}}+{\upalpha}_{n\;t{5}_{\mathrm{st}}}\ast t{5}_{\mathrm{st}}+{\upalpha}_{n\;\mathrm{cc}2{}_{\mathrm{st}}}\ast \mathrm{cc}{2}_{\mathrm{st}}+{\upalpha}_{\mathrm{n}\;\mathrm{s}2\mathrm{s}\mathrm{t}}\ast \mathrm{s}{2}_{\mathrm{st}} $$

Then, a logistic regression analysis for implantation as a dependent variable was conducted. The following independent variables were used in the analysis: six principal components (*f*_1_–*f*_6_), four levels of fragmentation (fr2–fr5), and age as an adjusted variable. The parameters of the chosen multivariate logistic regression model were used to create the ScPCA score, which can be treated as another predictor of implantation.

In the statistical analysis, the Kolmogorov-Smirnov test with the Lilliefors amendment and the Shapiro-Wilk test was used to verify the normality of distribution. Principal component analysis was conducted using the standardized morphokinetic parameters to obtain uncorrelated principal components. Univariate and multivariate logistic regression models were created. Based on the coefficients of the multivariate models, two prognostic parameters were built. The chi-squared test of independence was used to compare pregnancy rates between the studied groups. The Mann-Whitney *U* test was conducted to compare the prognostic parameter values between implantation and non-implantation groups. ROC analysis with the determination of area under the curve was carried out to check the effectiveness of created predictors. Statistical significance was determined at the *p* < 0.05 level. Statistical inference was conducted using Stata/IC 13.0 (Stata Corp. LP., College Station, TX, USA) and Statistica 12.5 (StatSoft, Tulsa, OK, USA).

## Results

The differences in the distribution of morphokinetic parameters between both the implanted and non-implanted groups (Fig. 1) are not as evident as in the case of embryos developed and not developed to the blastocyst stage [9]. Based on the algorithm proposed in [9], a multivariate logistic regression model, taking into account the parameters *t*2, *t*5, and cc2, was created. Using the coefficients of this model, the parameter Sc was created according to the following formula:

$$ \mathrm{S}\mathrm{c}=1.249\ast s\_t2+1.292\ast s\_\mathrm{t}5+1.231\ast s\_\mathrm{cc}2 $$

Then, the power of the constructed predictor was estimated. After dividing the embryos into four groups according to quartiles and the median value of the Sc parameter (C1–C4), statistically significant differences in pregnancy rates were found between the studied groups (*p* = 0.009) (Table 1).

**Table 1:** Pregnancy rates between quarters of the Sc parameter

Quarter (*N*)		C1 (118)	C2 (94)	C3 (115)	C4 (86)
Range		Sc ≤ 2.48	2.48 < Sc ≤ 3.73	3.73 < Sc ≤ 5.06	5.06 < Sc
Pregnancy rate	*N*	20	25	38	31
	%	16.9 %	26.6 %	33.0 %	36.0 %

Analyzing Sc values between the implanted and non-implanted groups also revealed statistically significant differences (*p* < 0.001). Sc values in the implanted group were significantly higher (Me = 4.98; *Q*_1_ = 2.50; *Q*_3_ = 5.08) than in the non-implanted group (Me = 3.73; *Q*_1_ = 1.29; *Q*_3_ = 5.02).

**Fig. 2:**
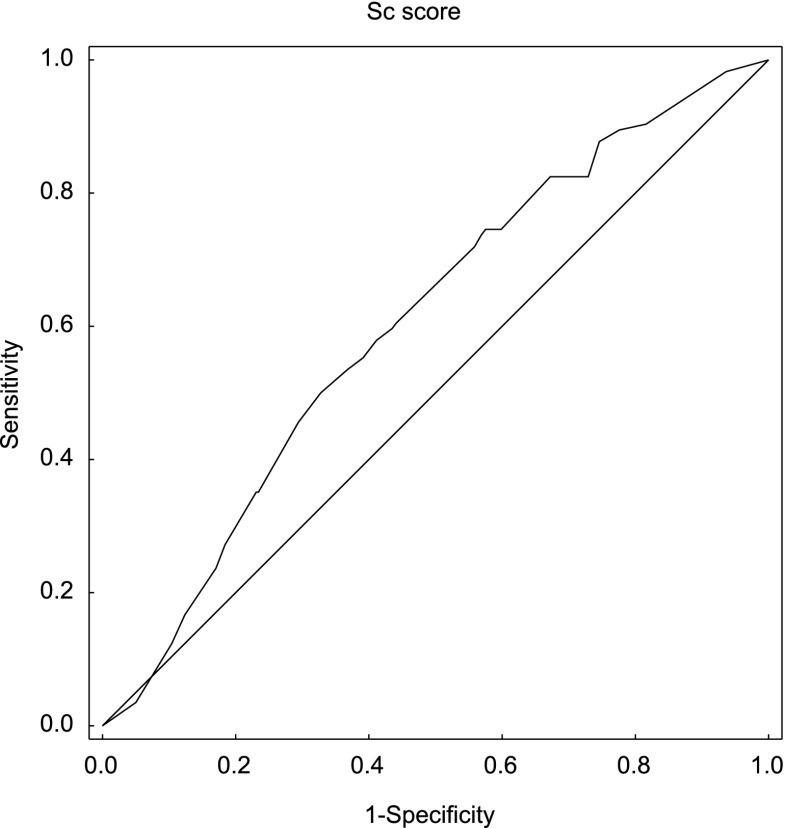
The ROC curve for implantation prediction by the Sc parameter (AUC = 0.61; 95 % CI 0.55–0.66)

The ROC curve created for the Sc parameter shows the quality of this predictor as a tool for identifying implantation (Fig. 2). The area under the ROC curve was AUC = 0.61, with a 95 % confidence interval (0.55, 0.66).

The created Sc predictor is statistically significant (the 95 % confidence interval does not include the 0.5 value), but its predictive power is considerably lower than in the case of the predictor for blastocyst formation presented in [9]. The strength of the considered predictor could be improved by the use of information contained in all morphokinetic parameters. But the crucial problem is that the parameters are strongly correlated with each other (Table 2), and this is in contrary to one of the assumptions of logistic regression analysis.

**Table 2:** Correlations between morphokinetic parameters

	*t*3_*m*_	*t*4_*m*_	*t*5_*m*_	cc2_*m*_	*s*2
*t*2_*m*_	0.58	0.62	0.33	0.19	NS
*t*3_*m*_		0.74	0.59	0.47	0.15
*t*4_*m*_			0.51	0.37	0.14
*t*5_*m*_				0.45	0.13
cc2_*m*_					0.21

To cope with this problem, the PCA method was used. Six new variables (*f*_1_–*f*_6_) called principal components, which are not correlated with each other, and contained the same information as the six morphokinetic parameters, were created. The matrix of coefficients of linear combinations for the principal components *f*_1_–*f*_6_ is presented in Table 3.

**Table 3:** Coefficients of the new factors obtained using the PCA method

	*f*_1_	*f*_2_	*f*_3_	*f*_4_	*f*_5_	*f*_6_
*t*2_st_	−0.363029	−0.577729	0.119448	0.503899	−0.481942	0.184363
*t*3_st_	−0.470337	−0.242126	0.212644	−0.104098	0.728281	0.365664
*t*4_st_	−0.461221	−0.105156	−0.569844	0.080034	0.169038	−0.645380
*t*5_st_	−0.420778	−0.015621	−0.046059	−0.780180	−0.441102	0.131638
cc2_st_	−0.369411	0.408948	0.702288	0.120262	−0.075756	−0.427653
*s*2_st_	−0.347490	0.655030	−0.347077	0.325115	−0.091951	0.464295

Univariate logistic regression analysis was performed for the created variables in order to evaluate their association with implantation. In addition to these parameters, the levels of fragmentation assessed in *t*2, *t*3, *t*4, and *t*5 time-points (fr2–fr5) were also included in the analysis. Because a woman’s age has a significant impact on the likelihood of becoming pregnant (pregnancy rate considerably decreases among older women [11]), the age of each woman was also included in the analysis as an adjusted variable. Univariate logistic regression results are shown in Table 4.

**Table 4:** Univariate logistic regression analysis in relation to implantation

Parameter	Coefficient	95 % confidence interval		*p* value
*f*_1_	0.243	0.092	0.394	0.002
*f*_2_	−0.097	−0.316	0.122	0.39
*f*_3_	0.006	−0.247	0.260	0.96
*f*_4_	0.076	−0.203	0.355	0.59
*f*_5_	0.187	−0.201	0.576	0.34
*f*_6_	0.208	−0.349	0.765	0.46
fr2	−0.533	−1.041	−0.025	0.04
fr3	−0.942	−1.567	−0.316	0.003
fr4	−0.990	−1.619	−0.361	0.002
fr5	−0.874	−1.492	−0.255	0.006
age	−0.136	−0.195	−0.078	<0.001

Among the principal components, only the first (*f*_1_) is significantly associated with implantation (*p* = 0.002). All levels of fragmentation are importantly (negatively) related with implantation, as well as the woman’s age (*p* < 0.001). Taking into account the variables whose significance was confirmed in univariate logistic regression, a multivariate logistic regression model was created (Table 5). This model takes into account the first principal component *f*_1_, the level of fragmentation assessed in the time *t*3, and the woman’s age. Based on the coefficients determined in the multivariate logistic regression model, parameter ScPCA was created as the sum of the products of the three parameters multiplied by the corresponding coefficients. It is described by the formula:

**Table 5:** Multivariate logistic regression model in relation to implantation

Parameter	Coefficient	95 % confidence interval		*p* value
*f*_1_	0.220	0.060	0.380	0.007
fr3	−0.783	−1.437	−0.130	0.02
age	−0.139	−0.199	−0.080	<0.001

$$ \mathrm{ScPCA}=0.22*{f}_1-0.783*\mathrm{f}\mathrm{r}3-0.139*\mathrm{age} $$

After transformation to the standardized morphokinetic parameters (coefficients from Table 3), the ScPCA parameter takes the form:

$$ \mathrm{ScPCA}=-0.08*t{2}_{\mathrm{st}}-0.103*t{3}_{\mathrm{st}}-0.101*t{4}_{\mathrm{st}}-0.092*t{5}_{\mathrm{st}}-0.081*\mathrm{cc}{2}_{\mathrm{st}}-0.076*s{2}_{\mathrm{st}}-0.783*\mathrm{f}\mathrm{r}3-0.139*\mathrm{age} $$

All the components of this formula have a negative sign, which means that all standardized morphokinetic parameters (the standardized distance from the median value), as well as the level of fragmentation and the woman’s age, are negatively associated with implantation. It follows that the ScPCA parameter has only negative values. The closer to zero it is, the greater the chance of pregnancy. The obtained coefficients can be interpreted as follows: increasing the age by 1 year decreases the ScPCA parameter by 0.139. For example, for the standardized *t*3 parameter, an increase by 1 decreases the ScPCA parameter by 0.103 (and similarly for the other morphokinetic parameters). Increasing the fragmentation by one category (in a four-point scale) decreases the ScPCA parameter by 0.783.

After dividing the embryos into four groups according to quartiles and the median value of the ScPCA parameter (C1–C4), statistically significant differences (*p* < 0.001) in pregnancy rates were found between the studied groups (Table 6). Pregnancy rate increases according to ScPCA rise. As can be seen in Table 6, in this study, it reached just over 12 % in the first quarter (C1) and about 46 % in the fourth (C4).

**Table 6:** Pregnancy rates between quarters of the ScPCA parameter

Quarter (*N*)		C1 (99)	C2 (100)	C3 (99)	C4 (100)
Range		ScPCA ≤ −6.11	−6.11 < ScPCA ≤ −5.52	−5.52 < ScPCA ≤ −5.02	−5.02 < ScPCA
Pregnancy rate	*N*	12	21	34	46
	%	12.1 %	21.0 %	34.3 %	46.0 %

**Fig. 3:**
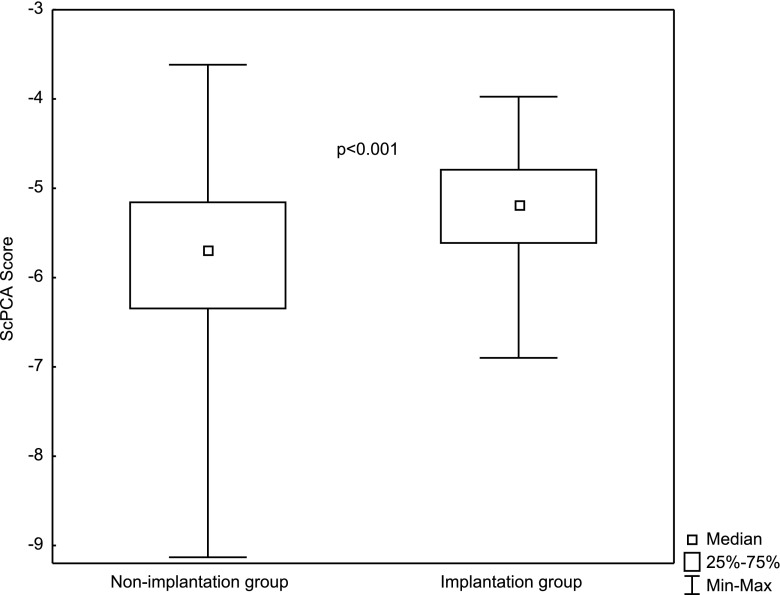
Differences in the ScPCA score (*p* < 0.001) between groups with and without implantation (median, quartiles, and min-max)

**Fig. 4:**
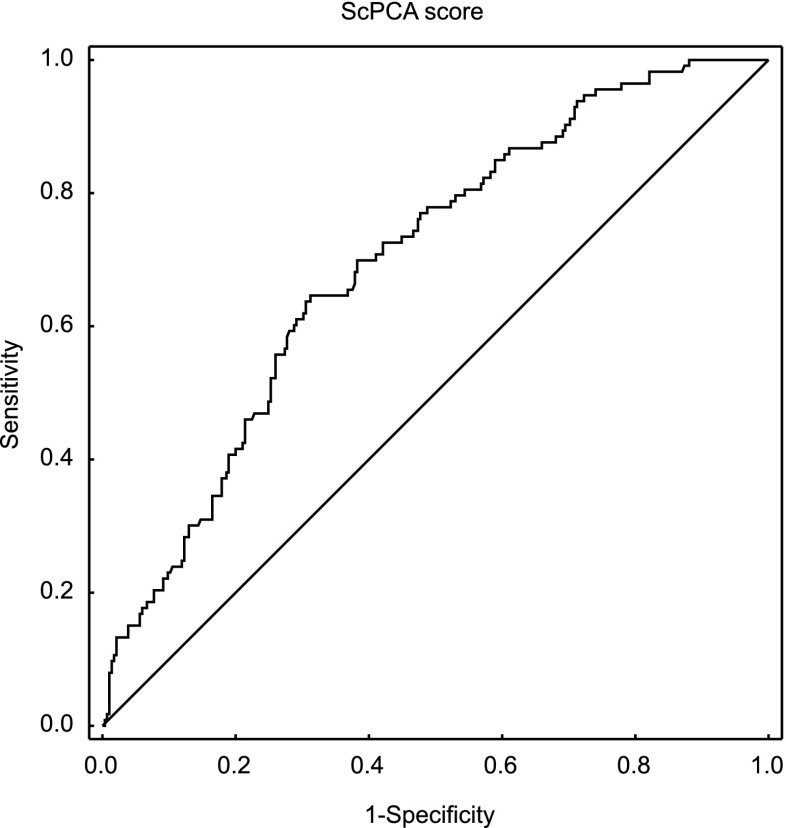
The ROC curve for implantation prediction by the ScPCA parameter (AUC = 0.70; 95 % CI 0.64–0.75)

Analyzing ScPCA values between the implantation and non-implantation groups also revealed statistically significant differences (*p* < 0.001). ScPCA values in the implantation group were significantly higher (Me = −5.18; *Q*_1_ = −5.61; *Q*_3_ = −4.79) than in the non-implantation group (Me = −5.69; *Q*_1_ = −6.34; *Q*_3_ = −5.16). These differences are presented in Fig. 3. The ROC curve created for the ScPCA parameter shows the high quality of this predictor as a tool for identifying implantation (Fig. 4). The area under the ROC curve was AUC = 0.70, with a 95 % confidence interval (0.64, 0.75). These results are much better than those gathered using the previous model based on the Sc parameter. The cutoff point ScPCA = −5.307 was determined using the minimal sum of squared coordinates method. Sensitivity for this cutoff point was 64.6 % and specificity 68.8 %.

The created model was validated on an independent data set containing 112 embryos, 40 of which implanted. The area under the ROC curve was very similar: AUC = 0.70, with a 95 % confidence interval (0.59, 0.80). Sensitivity for the determined cutoff point was 72.5 % and specificity 65.3 %. Just as shown in Fig. 3, ScPCA values in the group of implanted embryos were significantly (*p* < 0.001) higher (Me = −5.13; *Q*_1_ = −5.71; *Q*_3_ = −4.85) than in the group of non-implanted embryos (Me = −5.84; *Q*_1_ = −6.18; *Q*_3_ = −5.30).

## Discussion

The model for blastocyst formation presented in [9] has a high predictive power (the AUC value is greater than 0.8). However, the model created by an analogous procedure to predict embryo implantation (based on *t*2, *t*5, and cc2 variables) did not yield such promising results. After the division of the data into four quartiles according to the created parameter values, pregnancy rate changed from 16.9 % in the first quarter to 36 % in the fourth (Table 1). The area under the ROC curve for this parameter was AUC = 0.61, with a 95 % confidence interval (0.55, 0.66) (Fig. 2). The obtained results indicate that the constructed predictor is statistically significant, but its predictive power is not sufficient to effectively distinguish between embryos able and unable to implant. This may suggest that morphokinetic parameters are less important for predicting pregnancy than for predicting blastocyst formation, which is consistent with the information presented in Fig. 1 (small differences in the distribution of morphokinetic parameters between groups).

Many authors have confirmed the relationship between kinetic information and embryo implantation, but they do not specify any predictive models. Chamayou et al. [12] claim that time-lapse monitoring gives one the possibility to establish kinetic parameters predictive for implantation. They note the need to design a new embryo scoring system that must reflect embryo quality and its ability to implant, based not only on the times of cleavage but also on phenomena such as fragmentation, multinucleation, or asynchronous division. Similarly, Dal Canto et al. [13] declare that the ability of an embryo to implant is related with progressively earlier cleavage times during the first three mitosis cycles, and the analysis of its morphokinetic information may be helpful in choosing embryos more suitable for transfer. Lemmen et al. [14] indicate a synchrony in the appearance of nuclei after the first cleavage, degree of fragmentation, and blastomere evenness over time as features significantly associated with increased pregnancy success.

Some authors emphasize that we still know too little to fully trust the information contained in morphokinetic parameters. Kirkegaard et al. [15] state that several putative morphokinetic markers of embryo viability have been suggested in the literature, but the studies provide no unambiguous information with regard to what parameters are predictive. It remains to be explained whether embryo evaluation by time-lapse monitoring really improves pregnancy rates. In another work, Kirkegaard et al. [16] conclude that time-lapse parameters may not predict pregnancy. The authors found no difference in timing between implanted and non-implanted embryos, but an essential limitation of this study was the small size of analyzed groups (26 pregnant vs. 58 non-pregnant). Racowsky et al. [7] indicate that current evidence about time-lapse embryo imaging is still of very low quality, and further studies are necessary. They argue that the current evidence is insufficient to support the use of a time-lapse imaging system compared with a standard embryo selection system and that embryo selection based on time-lapse information should remain only an experimental strategy.

Aguilar et al. [17] present a predictive model for implantation based on three time-lapse parameters: the time of second polar body extrusion, time of pronuclear fading, and length of S-phase (the time during which the zygote’s DNA is replicated, i.e., from pronuclear appearance to pronuclear fading). Using these variables, they define a multivariate logistic regression model. An ROC analysis indicated that this model did not have a very high predictive power with regard to the probability of implantation (AUC = 0.605). It should be noted that the authors give the 95 % confidence interval for this value as (0.557–0.603), which is impossible because the upper limit of the confidence interval must be greater than the AUC value. Therefore, the reliability of the reported results appears to be limited.

The most popular morphokinetic model with a relatively high efficacy to identify embryos with pregnancy potential is proposed by Meseguer et al. in [18]. They propose a hierarchical, multivariable model that incorporates time-lapse information to classify embryos in accordance with their probability of implantation. The comparison of the presented hierarchical tree with categorizations based only on morphology showed that sorting effectiveness was better in the time-lapse categorization. This may suggest that it is possible to improve pregnancy rate by using morphokinetic information. However, the hierarchical nature of this model causes some limitations. The values of parameters are not treated “linearly” but are divided into compartments. Furthermore, the parameter considered earlier in the hierarchy has a much greater impact on the classification results than the parameter considered later. This could potentially reduce the predictive ability of the presented model. Freour et al. [19] evaluated the performance of the procedure published by Meseguer. They found that Meseguer’s model did not perform as well on their data as it did in the original publication. Freour appreciates the solution proposed by Meseguer and treats it as a fair basis for time-lapse embryo evaluation, but he states that such hierarchical models should not be universally used in any IVF clinic.

An alternative to hierarchical models are models of a linear nature, e.g., based on logistic regression analysis. The algorithm for the prediction of embryo implantation analogous to the one described in [9] gave unsatisfactory results, so we decided to create a model of a linear nature. To solve the problem of lack of monotonicity of morphokinetic variables, we transformed them into the absolute values of distance from the median. In order to use the time-lapse information as much as possible, we decided to incorporate all, both absolute (*t*2–*t*5) and relative (cc2, *s*2), morphokinetic parameters in the model. Because they were strongly correlated with each other (Table 2), and this is in contrary to one of the assumptions of logistic regression, we used principal component analysis—the data-mining method that creates new uncorrelated variables without the loss of contained information. The woman’s age was included in the analysis as an adjusted variable because it has a significant impact on pregnancy rate, particularly in older women [11]. In addition, because the estimation of the level of fragmentation forms part of almost all the schemes of embryo classification [20], we also included it in the analysis (assessed in *t*2–t5 time-points). The presence of fragments (fragment defined as an anuclear, membrane-bound extracellular cytoplasmic structure) has been found to be related to abnormalities in cell division that may indicate anomalies in chromosomal segregation [21]. Fujimoto et al. suggest that fragmentation may result from abnormalities in the oocyte membrane [22]. Using time-lapse monitoring of embryos and polarized light microscopy of the meiotic spindle, Stensen et al. showed that the fragmentation of embryos is associated with the progress of the meiotic and the mitotic cell cycles, the time between the first and the second mitosis and the duration of the third mitotic cell cycle [23].

After the division of the data into four quartiles according to the second created predictor (ScPCA) values, pregnancy rate changed from 12.1 % in the first quarter to 46 % in the fourth (Table 6). The area under the ROC curve for this parameter was AUC = 0.70, with a 95 % confidence interval (0.64, 0.75) (Fig. 4). Figure 3 shows that differences in ScPCA distribution between pregnancy and non-pregnancy groups are statistically significant. The created predictor has been validated on an independent data set, and the obtained results were very similar (AUC = 0.70). This shows that the model is reliable and repeatable and can become the basis for the creation of a more extensive model for application in clinical practice. Of course, other clinics may use other culture parameters, other media types, etc., which may result in slightly different division times. Therefore, the model should be understood in terms of the whole procedure, not only a final set of coefficients. The obtained coefficients of this model are valid for the particular clinic included in this study only. If one would like to use this model in another infertility treatment clinic, he or she should recalculate model parameters with respect to his or her clinic’s conditions. It is also important to keep in mind that despite the efforts of exploiting all possible information (linear nature of the model, application of the data-mining method), the power of the obtained model is less than in the case of prediction of blastocyst formation. This indicates that embryo implantation depends on more than morphokinetic factors; for example, on the receptivity of endometrium. The competent embryo and the receptive endometrium are two key components in successful embryo implantation [24]. Thorough study of factors associated with endometrial receptivity could allow for a significant increase in the power of prediction. Perhaps, such extended models would reach the prediction level of models for blastocyst formation or even exceed it.

## Conclusions

The created model shows that morphokinetic parameters contain information useful in the process of creating pregnancy prediction models. However, embryo quality (described by morphokinetic parameters) is not the only factor responsible for implantation. Therefore, this model’s power of prediction is not as high as in models for blastocyst formation. Important to note, however, is that the application of advanced data-mining methods allows one to extract more information from the data, and consequently to create more accurate and useful models.
